# The effects of ACEI/ARB, aldosterone receptor antagonists and statins on preventing recurrence of atrial fibrillation: A protocol for systematic review and network meta-analysis

**DOI:** 10.1097/MD.0000000000024280

**Published:** 2021-01-08

**Authors:** Qiulei Jia, Wenbing Han, Shuqing Shi, Yuanhui Hu

**Affiliations:** a Department of Cardiovascular, Guang’anmen Hospital, China Academy of Chinese Medical Sciences; b Emergency Department, Dongzhimen Hospital, Beijing University of Chinese Medicine, Beijing, China.

**Keywords:** ACEI/ARB, aldosterone receptor antagonists, atrial fibrillation, network meta-analysis, protocol, statins, systematic review

## Abstract

**Background:**

Atrial fibrillation (AF) is one of the most common arrhythmias, and is high relative to cardiovascular morbidity and mortality. AF-related complications and treatment costs bring about huge health burden, therefore the prevention recurrence of AF is imperative. “Upstream therapy” refers to the use of non-antiarrhythmic drugs (non-AADs) that modify the atrial substrate or target-specific mechanisms of AF to prevent the occurrence or recurrence of the arrhythmia. RAAS Blockers, aldosterone receptor antagonists and statins have an effect on preventing recurrence of atrial fibrillation. This protocol is designed for systematic review and network meta-analysis, which will assess comparative effects and safety of various non-antiarrhythmic drugs in preventing recurrence of atrial fibrillation.

**Methods:**

The Cochrane Library, MEDLINE, EMBASE, ClinicalTrials.gov will be searched from inception to Aug 31, 2020 to identify relevant studies. The Cochrane “Risk of bias” tool will be used to assess the methodological quality of eligible studies. The pair-wise meta-analysis will be performed by STATA 14.0 software. The network meta-analysis will be implemented in a Bayesian framework using Win BUGS 1.4.3 and the package “gemtc” V.0.8.1 of R-3.6.2 software. The network plots will be drawn using STATA 14.0 software. A comparison-adjusted funnel plot will be used to assess the publication bias using STATA 14.0 software. Quality of evidence will be assessed using the Grading of Recommendations Assessment, Development and Evaluation (GRADE) approach.

**Results:**

The results of this network meta-analysis will determine the preventive effect and rank ordering of these interventions for recurrence of AF. The report will follow the PRISMA checklist for network meta-analysis.

**Conclusion:**

This network meta-analysis will provide comprehensive evidence-based information in clinical practice.

**Inplasy registration number:**

INPLASY202090004.

## 1 Introduction

Atrial fibrillation (AF) is the most common arrhythmia and is associated with increased risk of all-cause mortality,^[1]^ stroke,^[2]^ heart failure, cognitive impairment,^[3]^ as well as decreased quality of life.^[4]^ The estimated numbers of adults with AF in Europe are 8 million, and about 3 to 5 million people are currently affected by AF.^[5,6]^ The annual incidence of AF in Asian is 5.38 per 1000 person-years.^[7]^ With the prolongation of life span, its prevalence is expected to double in the next 50 years.^[8]^ On the other hand, the management of patients with AF is costly. Direct-cost is about $2000 −14,200 per patient-year in the USA and between €450 to 3000 in Europe, which is equivalent to other chronic conditions such as diabetes.^[9]^ More than half of these expenses for AF are consisted of hospitalization costs, accounting for 52% to 72% of the total cost.^[10,11,12]^ In consideration its rising prevalence and substantial financial burden on health services, the prevention of AF and integrated management is the urgent need for healthcare systems. Catheter ablation is widely used in preventing recurrent AF,^[13]^ but it is difficult to predict the rhythm outcome of the patients undergoing catheter ablation therapy.^[14,15]^ Severe complications of catheter ablation occurred in 5% to 7% of patients, and 2% to 3% will experience life-threatening but usually manageable complications,^[16,17,18,19]^ thus pharmacotherapy is still the essential treatment for atrial fibrillation.

“Upstream therapy” was first proposed in 2010 ESC Guidelines for the management of atrial fibrillation, which refers to the use of non-antiarrhythmic drugs (non-AADs) that modify the atrial substrate or target-specific mechanisms of AF to prevent the occurrence or recurrence of the arrhythmia.^[20]^ It is generally known that AF can lead to electrical modifications and structural atrial remodeling represented essentially by a shortening and dispersion of the action potential, atrial enlargement and fibrosis, which make the restoration of the sinus rhythm and its subsequent maintenance more difficult. Upstream therapy aims to counter and/or delay structural atrial remodelling, such as fibrosis, hypertrophy, inflammation, oxidative stress, but effects on atrialion channels, gap junctions, and calcium handling are also evident.^[21]^ As the research on upstream therapy of AF constantly goes deeper, the main research focuses on angiotensin-converting enzyme inhibitors (ACEI), angiotensin receptor block (ARB), mineralocorticoid receptor antagonist (MRAS) and statins.^[22]^

Activation of the renin-angiotensin-aldosterone system (RAAS) promotes structural atrial remodeling, and angiotensin II is up-regulated in AF.^[23,24]^ Aldosterone causes a substrate for atrial arrhythmias characterized by atrial fibrosis, myocyte hypertrophy, and conduction disturbances.^[25]^ It is reported that a decrease in aldosterone levels have been reported in the AF patients with longer sinus rhythm maintenance after successful cardioversion.^[26]^ Statins have anti-inflammatory and antioxidant effects that can prevent atrial remodeling.^[27]^ Evidence from animal studies showed that simvastatin decreases atrial fibroblast proliferation and reduces electric remodeling.^[28,29]^

There have been several systematic reviews demonstrated the preventive effect of ACEIs, ARBs, MRAs and statins on recurrent AF,^[30,31,32]^ whose controlled intervention was either placebo or conventional treatment. Traditional systematic reviews present pairwise “head to head” comparisons, which cannot compare the effect of multiple treatments simultaneously, making it difficult to assess which interventions work best. Network meta-analysis (NMA) can be used to undertake both direct and indirect comparisons of various interventions and rank ordering of the interventions, covering the shortage of pair-wise analysis. Therefore, we plan to conduct the systematic review and NMA to assess comparative effects and safety of these non-antiarrhythmic drugs in preventing recurrence of atrial fibrillation.

## 2 Methods

### 2.1 Study design

This network meta-analysis has been prospectively registered with the International Platform of Registered Systematic Review and Meta-Analysis Protocols (INPLASY) (INPLASY202090004 Available from https://inplasy.com/inplasy-2020-9-0004/). A protocol for systematic review and Bayesian NMA will be performed following the Preferred Reporting Items for Systematic Review and Meta-Analysis Protocols guideline.^[33]^

### 2.2 Criteria for considering studies for this review

#### 2.2.1 Types of studies

Only randomized controlled trials (RCTs) reported as comparing ACEI/ARB, aldosterone receptor antagonists, or statin combined with standard therapy for AF with placebo, or no drug combined with standard therapy will be included in this study. We will only include parallel-group trials, cluster-randomized trials, and cross-over trials will be excluded.

#### 2.2.2 Types of participants

Patients aged 18** **years or older, of both sexes, diagnosed with paroxysmal AF or persistent AF treated with electrical cardioversion will be included. The diagnostic criteria for AF will be referred to the 2016 European society of cardiology criteria for the clinical diagnosis of AF^[34]^ and the AHA/ACC/HRS 2019 guidelines for the management of patients with AF.^[35]^

#### 2.2.3 Types of interventions

We will include studies comparing the effects of ACEI/ARB, aldosterone receptor antagonists and statins combined with standard therapy for AF (rate control, anticoagulants and antiarrhythmic) respectively with placebo, or no drug combined with standard therapy. Studies that compared different dosages of any intervention above will be excluded. The drug in combination with standard therapy will not contain ACEI/ARB, aldosterone receptor antagonists or statins.

#### 2.2.4 Types of outcome measures

We will include studies that reported at least one of the following outcomes.

The primary outcome is the time to a first electrocardiographically confirmed recurrence of atrial fibrillation. The secondary outcomes are

1. left atrial dimension in echocardiography;
2. adverse drug events/reactions (ADEs/ADRs).

### 2.3 Search methods for identification of studies

Searches for published RCTs will be undertaken in MEDLINE, EMBASE, and Cochrane Central Register of Controlled Trials (CENTRAL). We will screen reference lists of previously published meta-analyses. We will also undertake searches for published, unpublished and ongoing studies in ClinicalTrials.gov. Including unpublished data may decrease publication bias. The following search terms will be used: “atrial fibrillation”, “Angiotensin-Converting Enzyme Inhibitors”, “Angiotensin Receptor Antagonists”, “Hydroxymethylglutaryl-CoA Reductase Inhibitors”, and “Mineralocorticoid Receptor Antagonists”. Our detailed search strategy for the different databases is outlined in Tables 1–3. Because of the language barrier, the search will be restricted to trials published in English. There are no restrictions on the types of study.

**Table 1:** Search criteria for the systematic review: MEDLINE.

Step	Keywords **(**including MeSH words**)**
#1	(atrial fibrillation [MeSH Terms]) OR (Atrial Fibrillations) OR (Fibrillation, Atrial) OR (Fibrillations, Atrial) OR (Auricular Fibrillation) OR (Auricular Fibrillations) OR (Fibrillation, Auricular) OR (Fibrillations, Auricular) OR (Persistent Atrial Fibrillation) OR (Atrial Fibrillation, Persistent) OR (Atrial Fibrillations, Persistent) OR (Fibrillation, Persistent Atrial) OR (Fibrillations, Persistent Atrial) OR (Persistent Atrial Fibrillations) OR (Familial Atrial Fibrillation) OR (Atrial Fibrillation, Familial) OR (Atrial Fibrillations, Familial) OR (Familial Atrial Fibrillations) OR (Fibrillation, Familial Atrial) OR (Fibrillations, Familial Atrial) OR (Paroxysmal Atrial Fibrillation) OR (Atrial Fibrillation, Paroxysmal) OR (Atrial Fibrillations, Paroxysmal) OR (Fibrillation, Paroxysmal Atrial) OR (Fibrillations, Paroxysmal Atrial) OR (Paroxysmal Atrial Fibrillations)
#2	(Angiotensin-Converting Enzyme Inhibitors [MeSH Terms]) OR (Angiotensin Converting Enzyme Inhibitors) OR (Inhibitors, Kininase II) OR (Kininase II Antagonists) OR (Kininase II Inhibitors) OR (Angiotensin I-Converting Enzyme Inhibitors) OR (Angiotensin I Converting Enzyme Inhibitors) OR (Antagonists, Angiotensin-Converting Enzyme) OR (Antagonists, Angiotensin Converting Enzyme) OR (Antagonists, Kininase II) OR (Inhibitors, ACE) OR (ACE Inhibitors) OR (Inhibitors, Angiotensin-Converting Enzyme) OR (Enzyme Inhibitors, Angiotensin-Converting) OR (Inhibitors, Angiotensin Converting Enzyme) OR (Angiotensin-Converting Enzyme Antagonists) OR (Angiotensin Converting Enzyme Antagonists) OR (Enzyme Antagonists, Angiotensin-Converting)
#3	(Angiotensin Receptor Antagonists [MeSH Terms]) OR (Antagonists, Angiotensin Receptor) OR (Receptor Antagonists, Angiotensin) OR (Angiotensin Receptor Blockers) OR (Receptor Blockers, Angiotensin) OR (Angiotensin II Receptor Antagonists) OR (Angiotensin II Receptor Blockers)
#4	(Hydroxymethylglutaryl-CoA Reductase Inhibitors[MeSH Terms]) OR (Hydroxymethylglutaryl CoA Reductase Inhibitors) OR (Inhibitors, Hydroxymethylglutaryl-CoA Reductase) OR (Reductase Inhibitors, Hydroxymethylglutaryl-CoA) OR (Inhibitors, HMG-CoA Reductase) OR (Inhibitors, HMG CoA Reductase) OR (Reductase Inhibitors, HMG-CoA) OR (HMG-CoA Reductase Inhibitors) OR (HMG CoA Reductase Inhibitors) OR (Statins, HMG-CoA) OR (HMG-CoA Statins) OR (Statins, HMG CoA) OR (Inhibitors, Hydroxymethylglutaryl-CoA) OR (Hydroxymethylglutaryl-CoA Inhibitors) OR (Inhibitors, Hydroxymethylglutaryl CoA) OR (Statins) OR (Inhibitors, Hydroxymethylglutaryl-Coenzyme A) OR (Inhibitors, Hydroxymethylglutaryl Coenzyme A) OR (Hydroxymethylglutaryl-Coenzyme A Inhibitors)
#5	(Mineralocorticoid Receptor Antagonists [MeSH Terms]) OR (Antagonists, Mineralocorticoid Receptor) OR (Receptor Antagonists, Mineralocorticoid) OR (Mineralocorticoid Antagonists) OR (Antagonists, Mineralocorticoid) OR (Aldosterone Receptor Antagonists) OR (Antagonists, Aldosterone Receptor) OR (Receptor Antagonists, Aldosterone) OR (Aldosterone Antagonists) OR (Antagonists, Aldosterone)
#6	#1 and #2
#7	#1 and #3
#8	#1 and #4
#9	#1 and #5
#10	#6 or #7 or #8 or #9

**Table 2:** Search criteria for the systematic review: EMBASE.

Step	Keywords **(**including MeSH words**)**
#1	“atrial fibrillation”/exp
#2	“atrium fibrillation”: ab,ti OR “auricular fibrillation”: ab,ti OR “auricular fibrillation”: ab,ti OR “cardiac atrial fibrillation”: ab,ti OR “cardiac atrium fibrillation”: ab,ti OR “fibrillation, heart atrium”: ab,ti OR “heart atrial fibrillation”: ab,ti OR “heart atrium fibrillation”: ab,ti OR “heart fibrillation atrium”: ab,ti OR “non-valvular atrial fibrillation”: ab,ti OR “nonvalvular atrial fibrillation”: ab,ti
#3	“dipeptidyl carboxypeptidase inhibitor”/exp
#4	“ace inhibitor”: ab,ti OR “angiotensin converting enzyme inhibiting agent”: ab,ti OR “angiotensin converting enzyme inhibitor”: ab,ti OR “angiotensin converting enzyme inhibitors”: ab,ti OR “angiotensin i converting enzyme inhibitor”: ab,ti OR “angiotensin-converting enzyme inhibitors”: ab,ti OR “converting enzyme inhibitor”: ab,ti OR “dipeptidyl carboxypeptidase i inhibitor”: ab,ti OR “kininase ii inhibitor”: ab,ti OR “peptidyl dipeptidase inhibitor”: ab,ti OR ’peptidyldipeptide hydrolase inhibitor’:ab,ti
#5	“angiotensin receptor antagonist”/exp
#6	“angiotensin ii receptor antagonist”: ab,ti OR “angiotensin ii receptor antagonists”: ab,ti OR “angiotensin ii receptor blocker”: ab,ti OR “angiotensin ii receptor blockers”: ab,ti OR “angiotensin ii receptor blocking agent”: ab,ti OR “angiotensin ii receptor blocking agents”: ab,ti OR “angiotensin receptor antagonists”: ab,ti OR “angiotensin receptor blocker”: ab,ti OR “angiotensin receptor blockers”: ab,ti OR “angiotensin receptor blocking agent”: ab,ti OR “angiotensin receptor blocking agents”: ab,ti
#7	“hydroxymethylglutaryl coenzyme a reductase inhibitor”/exp
#8	“hmg coa reductase inhibitor”: ab,ti OR “hmg coa reductase inhibitors”: ab,ti OR “hmg coenzyme a reductase inhibitor”: ab,ti OR “hmg-coa reductase inhibitors”: ab,ti OR “hydroxymethylglutaryl coa reductase inhibitors”: ab,ti OR “hydroxymethylglutaryl-coa reductase inhibitors”: ab,ti OR (statin: ab,ti AND drug: ab,ti) OR statins: ab,ti OR vastatin: ab,ti
#9	“mineralocorticoid antagonist”/exp
#10	“antimineralocorticoid”: ab,ti OR “mineralocorticoid receptor antagonists”: ab,ti
#11	#1 OR #2
#12	#3 OR #4
#13	#5 OR #6
#14	#7 OR #8
#15	#9 OR #10
#16	#11 AND #12
#17	#11 AND #13
#18	#11 AND #14
#19	#11 AND #15
#20	#16 or #17 or #18 or #19
#21	#20 AND [embase]/lim NOT [medline]/lim

**Table 3:** Search criteria for the systematic review: The Cochrane Library.

Step	Keywords **(**including MeSH words**)**
#1	(auricular fibrillation): ti,ab,kw OR (atrium fibrillation):ti,ab,kw OR (auricular fibrilation): ti,ab,kw (Word variations have been searched)
#2	atrial fibrillation
#3	#1 or #2
#4	MeSH descriptor: [Angiotensin-Converting Enzyme Inhibitors] explode all trees
#5	(Angiotensin I Converting Enzyme Inhibitors): ti,ab,kw OR (Kininase II Antagonists): ti,ab,kw OR (Kininase II Inhibitors):ti,ab,kw OR (ACE Inhibitors): ti,ab,kw OR (Angiotensin Converting Enzyme Antagonists): ti,ab,kw (Word variations have been searched)
#6	#4 or #5
#7	MeSH descriptor: [Angiotensin Receptor Antagonists] explode all trees
#8	(Angiotensin Receptor Blockers): ti,ab,kw OR (Antagonists, Angiotensin Receptor): ti,ab,kw OR (Angiotensin II Receptor Antagonists): ti,ab,kw OR (Angiotensin II Receptor Blockers): ti,ab,kw (Word variations have been searched)
#9	#7 or #8
#10	(HMG CoA Reductase Inhibitors): ti,ab,kw OR (Hydroxymethylglutaryl CoA Reductase Inhibitors): ti,ab,kw OR (Hydroxymethylglutaryl-Coenzyme A Inhibitors):ti,ab,kw OR (Inhibitors, Hydroxymethylglutaryl-CoA): ti,ab,kw OR (Statins): ti,ab,kw (Word variations have been searched)
#11	MeSH descriptor: [Hydroxymethylglutaryl-CoA Reductase Inhibitors] explode all trees
#12	#10 or #11
#13	MeSH descriptor: [Mineralocorticoid Receptor Antagonists] explode all trees
#14	(Aldosterone Antagonists): ti,ab,kw OR (Antagonists, Mineralocorticoid): ti,ab,kw OR (Aldosterone Receptor Antagonists): ti,ab,kw OR (Receptor Antagonists, Mineralocorticoid): ti,ab,kw OR (Mineralocorticoid Antagonists): ti,ab,kw (Word variations have been searched)
#15	#13 or #14
#16	#3 AND #6
#17	#3 AND #9
#18	#3 AND #12
#19	#3 AND #15
#20	#16 OR #17 OR #18 OR #19

### 2.4 Data collection and analysis

#### 2.4.1 Selection of studies

Three researchers (JQL, HWB, and SSQ) will independently scan titles and abstracts retrieved by the search after duplicate removal. A trial will be excluded if both researchers agree that it does not meet the inclusion criteria. Full text of articles would be retrieved for further assessment if the information met the inclusion criteria. Any disagreement between reviewers will be resolved by discussion or consulting a third party (HYH).

#### 2.4.2 Data extraction and management

Data concerning details of the study population, intervention and outcomes will be extracted independently by 2 researchers (JQL and HWB). For binary outcomes, the number of events and total number in each group will be extracted. For continuous outcomes, mean, standard deviation (SD) and sample size of each group will be extracted. The data extraction form included the following items:

1. General information: title, authors, and year of publication.
2. Trial characteristics: study design, method of randomization, allocation concealment, blinding.
3. Patients: number in treatment and control groups, age, diagnostic criteria, withdrawals/losses to follow-up (reasons/description), subgroups.
4. Intervention: intervention (dose, course of treatment, and frequency), comparison intervention (dose, course of treatment, and frequency).
5. Outcomes: outcomes specified above.

#### 2.4.3 Outcome data

For continuous outcomes, we will extract means, SDs and the number of patients randomized in each study arm. When means and their SDs are unavailable, we will contact study authors to provide the data. When standard errors, t-statistics or *P* values are reported, these will be transformed to SDs. If neither of the measures mentioned above is reported in the original report or a previous systematic review, the mean value of known SDs will be calculated from the group of included studies.^[36]^ For dichotomous outcomes, we will extract the number of patients experiencing any adverse event /reactions (ADEs/ADRs) out of the total number of treated patients.

#### 2.4.4 Missing outcome data

Missing outcome data are a common problem in clinical trials and systematic reviews, as it lowers credibility by potentially biasing the results. Firstly, we will identify the reason why the outcome data may be missing, because the risk of bias due to missing data depends on the missing data mechanism. The mechanisms of missing data are divided as missing completely at random (MCAR), missing at random (MAR), and missing not at random (MNAR) or informatively missing. When missing data are MCAR or MAR, biased estimate is negligible. When the mechanism is MNAR, which means that the reasons for dropout are associated with the actual effect of the intervention, biased estimate is non-negligible.^[37]^ Last observation carried forward (LOCF) will be used to deal with missing data. The method conforms with the intention-to-treat principle. To obtain missing data, we will contact the authors by emails. Otherwise, the data will be verified from other trials in the network or other published meta-analyses.^[36]^

### 2.5 Assessment of risk of bias in included studies

The methodological quality of trials will be assessed independently using criteria from the Cochrane Handbook for Systematic Review of Interventions, Version 5.1.0.^[38]^ Seven domains are considered such as sequence generation (selection bias), allocation concealment (selection bias), blinding of participants and personnel (performance bias), blinding of outcome assessment (detection bias), incomplete outcome data (attrition bias), selective outcome reporting (reporting bias), and other bias. Three levels of “low risk”, “high risk”, or “unclear risk” are the quality appraisal category. Any disagreements will be resolved by mutual consensus.

### 2.6 Statistical analysis

We will perform Bayesian NMAs to compare the effects of different interventions. We will conduct both standard meta-analyses of each pair-wise direct comparison between interventions and a network meta-analysis combining results of all these comparisons in 1 analysis, exploiting both the direct comparisons within trials and the indirect comparisons across trials for each outcome.

#### 2.6.1 Pairwise meta-analysis

Pairwise meta-analyses will be performed using Stata 14.0 software (Stata Corp, College Station, Tex). Odds ratio (OR) with 95% CI will be used for the incidence of adverse drug events/reactions (ADEs/ADRs). Mean differences or standard mean differences (SMDs) with 95% CI will be used for the time to a first electrocardiographically confirmed recurrence of atrial fibrillation and left atrial dimension in echocardiography. We will assess clinical and methodological heterogeneity by means of examination of the characteristics of the included trials. Chi-Squared tests for heterogeneity will be used to assess between study heterogeneity for each outcome, and the extent of the observed heterogeneity in effect sizes between studies will be assessed by the *I*^2^. If the *P* value is ≥.1 and *I*^2^ ≤ 50%, which suggests there is no statistical heterogeneity, then the fixed effects model will be employed. If the *P* value is <.1 and *I*^2^ > 50%, the random-effects model will be used. When heterogeneity among studies is obvious (*I*^2^ > 50%), the sources of heterogeneity will be investigated using subgroup analysis, sensitivity analysis, and meta-regression analysis. If quantitative synthesis cannot be undertaken, we will conduct qualitative analysis.

#### 2.6.2 Network meta-analysis

The network meta-analysis will be implemented in a Bayesian framework using Open BUGS 1.4.3 (MRC Biostatistics Unit, Cambridge, UK) and the package “gemtc” V.0.8.1 of R-3.6.2 software. The Markov Chains Monte Carlo sampler will be used to generate samples. A total of 5000 simulations for each chain will be set as the “burn-in” period. Then, posterior summaries will be based on 100,000 subsequent simulations. The results of the network meta-analysis will be reported as ORs or SMD with 95% CIs. To rank the treatments for each outcome, we will use the surface under the cumulative ranking curve (SUCRA) and the mean ranks.^[39]^ SUCRA values would be 100% when a treatment is certain to be the best, and 0% when a treatment is certain to be the worst.^[39]^ A node splitting method will be used to examine the inconsistency between direct and indirect comparisons when a loop connecting 3 arms exists.^[40]^ The network plot will be drawn by Stata 14.0 software to present the all pairwise comparisons between the eligible interventions. Nodes in network plot represent different interventions and edges represent direct comparisons between the 2 interventions. The size of nodes and thickness of edges are associated with sample sizes and numbers of RCTs respectively.

#### 2.6.3 Measures for transitivity assumption

The transitivity assumption underlying network meta-analysis will be assessed by comparing the distribution of clinical and methodological variables that can act as effect modifiers across treatment comparisons.^[41]^ Clinical variables include baseline characteristics of patients, interventions and comparisons, follow-up time and clinical outcomes. Methodological factors refer to the design and quality of each RCT.

Subgroup analysis and network meta-regression analysis will be conducted according to the following characteristics:

1. type of AF (paroxysmal AF or permanent AF);
2. course of disease;
3. combined disease (hypertension, diabetes or coronary heart disease, etc.);
4. types of interventions;
5. ethnic population.

We will perform the following sensitivity analyses to evaluate the robustness of the model:

1. Analysing only studies with low risk of bias;
2. Analysing only patients with paroxysmal AF or permanent AF;
3. Analysing only patients average age >60 years old.

A comparison-adjusted funnel plot will be used to assess the publication bias and small-study effects using STATA 14.0 software.^[42]^

### 2.7 Assessment of the quality of evidence

The quality of evidence will be assessed using The Grading of Recommendations Assessment, Development and Evaluation (GRADE) Working Group by which a determination of high, moderate, low, or very low will be made for each major outcome. The quality rating may be rated down for risk of bias, inconsistency, indirectness, imprecision, publication bias.^[43]^

## 3 Discussion, ethics and dissemination

The existing systematic reviews have shown the effectiveness of ACEI/ARB, stains, and MRA in the prevention of AF recurrent, but it lacks studies which directly compare among these different methods. Therefore, NMA will be used to compare the difference in effectiveness among the various therapies, to provide reliable evidence-based medical evidence for the upstream therapy for AF. We will publish the results of this review in peer-reviewed publications, and the data set will be made freely available.

In terms of advantages, we will comprehensively search a substantial amount of data from both published and unpublished studies from journal articles, academic dissertation, clinical study reports, ongoing clinical trials. GRADE approach will be used to evaluate the quality of evidence derived from the included studies. Furthermore, we will provide a relative ranking of the effects on preventing AF recurrent, which can help clinicians make evidence-based decisions. The different type of AF, course of the disease and combined disease may have an impact on the treatment effect, so we will use subgroup analysis and meta-regression analysis to reduce the inconsistency or heterogeneity.

However, there are several limits in our study:

1. Only English literature will be included in this study, which will lead to selection bias.
2. According to the inclusion criteria we established through preliminary screening literature, there is no study comparing different therapies in head-to-head trials, therefore findings will be mostly based on indirect trial comparisons.

This review does not require ethical approval. The framework of the PRISMA statements for NMA will be applied to guide review authors to perform this study. The results will be disseminated by a peer-reviewed publication.

## Author contributions

JQL, HWB, and SSQ planed and designed the study; HYH tested the feasibility of the study; JQL and HWB wrote the manuscript; all authors approved the final version of the manuscript.

**Conceptualization:** Yuanhui Hu.

**Data curation:** Qiulei Jia, Shuqing Shi.

**Methodology:** Qiulei Jia, Wenbing Han, Shuqing Shi.

**Supervision:** Yuanhui Hu.

**Writing – original draft:** Qiulei Jia.

**Writing – review & editing:** Wenbing Han.
